# Supplementary material for: Role of miR-181c in Diet-induced obesity through regulation of lipid synthesis in liver
Source: PLoS One. 2021 Dec 8;16(12):e0256973. doi: 10.1371/journal.pone.0256973 (PMC8654194; doi:10.1371/journal.pone.0256973)
Supplement: S1 Fig — (PPTX) [file pone.0256973.s001.pptx]

## Slide 1
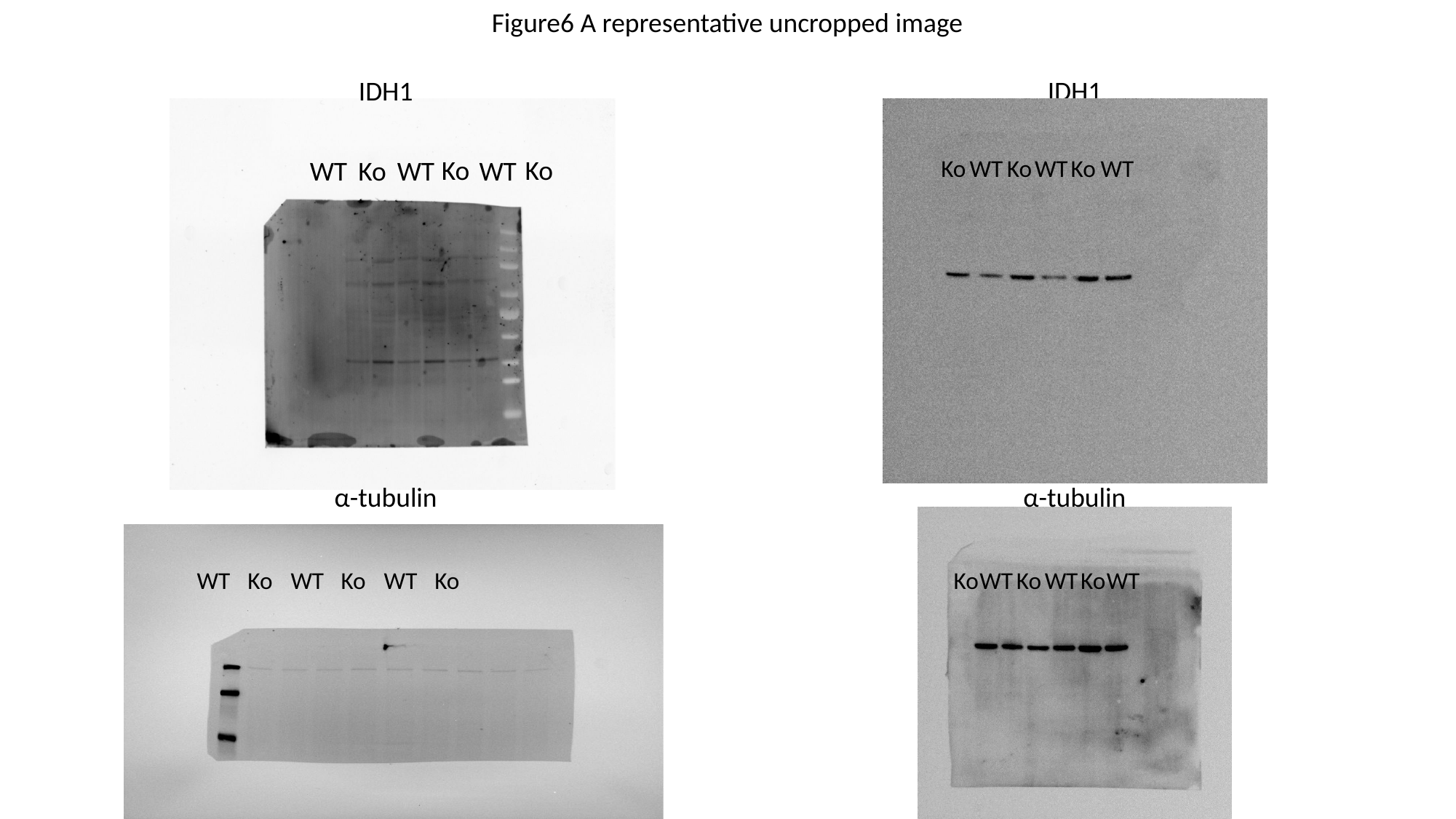

Figure6 A representative uncropped image
IDH1
IDH1
Ko
WT
Ko
WT
Ko
WT
Ko
Ko
WT
Ko
WT
WT
α-tubulin
α-tubulin
WT
Ko
WT
Ko
WT
Ko
Ko
WT
Ko
WT
Ko
WT

## Slide 2
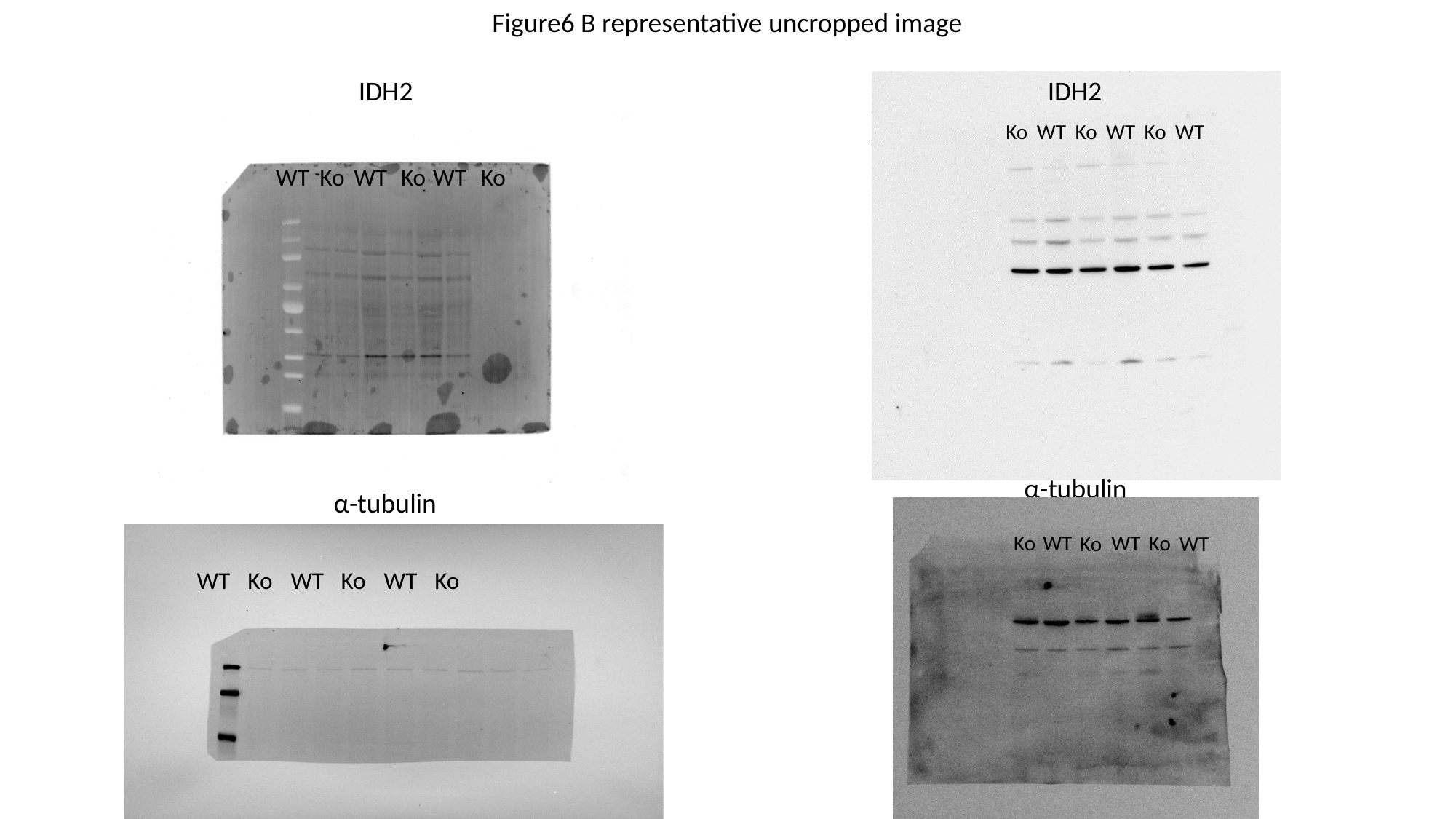

Figure6 B representative uncropped image
IDH2
IDH2
Ko
Ko
Ko
WT
WT
WT
WT
Ko
WT
Ko
WT
Ko
α-tubulin
α-tubulin
WT
Ko
Ko
WT
Ko
WT
WT
Ko
WT
Ko
WT
Ko
