# Supplementary material for: Role of miR-181c in Diet-induced obesity through regulation of lipid synthesis in liver
Source: PLoS One. 2021 Dec 8;16(12):e0256973. doi: 10.1371/journal.pone.0256973 (PMC8654194; doi:10.1371/journal.pone.0256973)
Supplement: S2 Fig — (PPTX) [file pone.0256973.s002.pptx]

## Slide 1
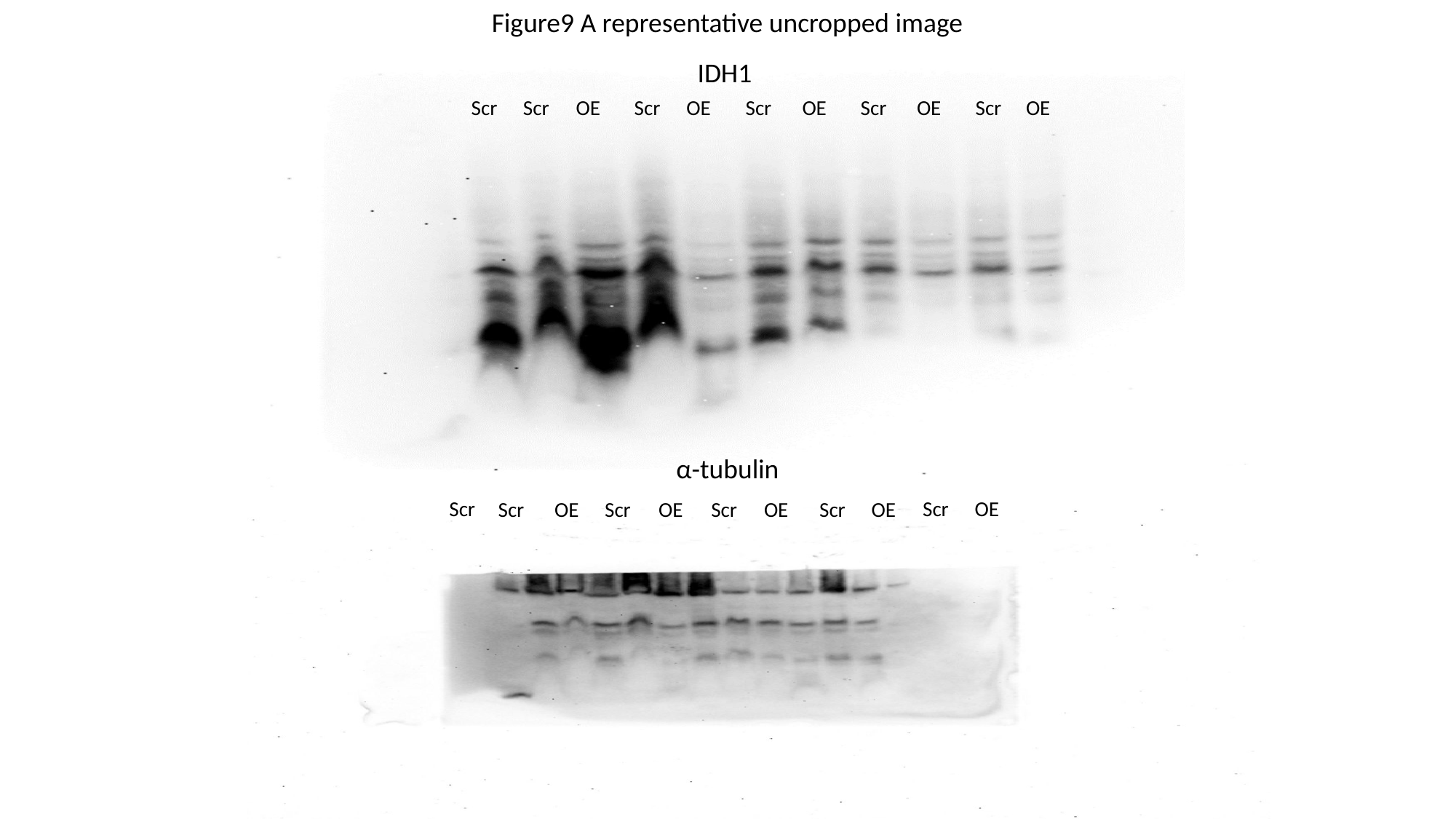

Figure9 A representative uncropped image
IDH1
OE
OE
OE
OE
OE
Scr
Scr
Scr
Scr
Scr
Scr
α-tubulin
Scr
Scr
OE
OE
Scr
OE
Scr
OE
Scr
OE
Scr

## Slide 2
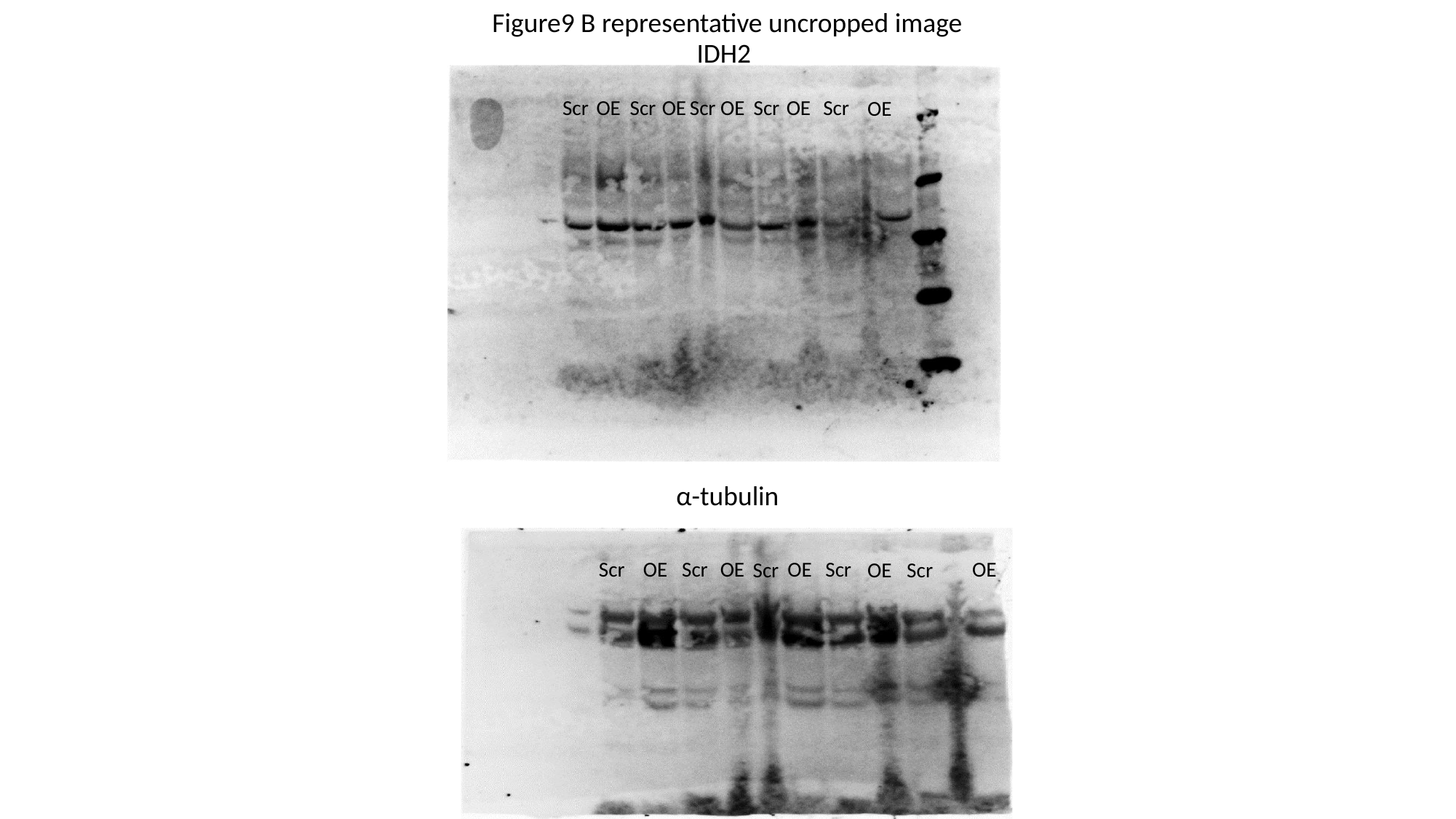

Figure9 B representative uncropped image
IDH2
Scr
OE
Scr
Scr
OE
Scr
OE
Scr
OE
OE
α-tubulin
OE
Scr
OE
Scr
OE
Scr
OE
OE
Scr
Scr
